# A Measurement Solution of Face Gears with 3D Optical Scanning

**DOI:** 10.3390/ma15176069

**Published:** 2022-09-01

**Authors:** Xinxin Lu, Xing Zhao, Bo Hu, Yuansheng Zhou, Zhezhen Cao, Jinyuan Tang

**Affiliations:** 1 State Key Laboratory of High Performance Complex Manufacturing, Central South University, Changsha 410083, China; 2 College of Mechanical and Electrical Engineering, Central South University, Changsha 410083, China

**Keywords:** face gears, 3D optical scanning, point clouds, tooth surface deviations, loaded tooth contact analysis

## Abstract

Gears are usually measured by the contact metrology method in gear measuring centers or coordinate measuring machines. Recently, three-dimensional (3D) optical scanning, a non-contact metrology method, has been applied in the industry as an advanced measurement technology mainly due to its high efficiency. However, its applications to gears with complicated geometry, such as face gears, are still limited due to its relatively low accuracy and the void of related measurement solutions. In this work, an accurate measurement solution with 3D optical scanning is proposed for the tooth surface deviations of orthogonal face gears. First, point cloud collection is carried out by the 3D scanner. Furthermore, the measurement solution is implemented with a three-stage algorithm by aligning point clouds with the design model. Subsequently, 3D modeling is studied by numbering the points and reconstructing the real tooth surfaces. An example with a measurement experiment and loaded tooth contact analysis is given to show the validity of the proposed method.

## 1. Introduction

Face gears [1,2,3,4] have been applied in some transmission systems due to the advantages of smooth transmission, large coincidence, and small footprint. Compared with the bevel gear drives, face gear drives have smaller axial forces and lower sensitivity to axial installation errors. Moreover, the support apparatus of face gear drives can be designed in a relatively more compact way than spiral bevel gear drives, and it can significantly save weight. The above strengths make face gears receive attention and research. Some car companies, such as Audi, have developed the advanced technology of face gears and applied it to automobile differentials. In the aerospace industry, face gears have been successfully used in helicopter transmission systems. More types of face gears are studied nowadays. According to the macroscopic form of the tooth surface, the types of face gears can be divided into spur face gears, helical face gears, conical face gears, worm face gears, asymmetric helical face gears [5,6], and so on. The performance of face gear drives is constantly improved, and the application scenarios are wider.

In terms of processing, face gears can be machined by grinding, CNC plunge milling, hobbing, and other processing methods [7,8,9]. The spur face gears, a simple type of face gears, can reach the desired accuracy level in the aerospace industry by grinding with “grinding-measurement-correction” [10,11]. The precision grade is evaluated by the measurement, so the reliable measurement of face gears is the basis of the correction of the machine tool settings in the processing. The measurement method of face gears generally adopts the contact measuring machines [12,13,14], such as coordinate measuring machines (CMMs) and gear measuring centers (GMCs). CMMs are universal measuring tools and can measure various workpieces. GMCs are specialized machines for measuring gears, which are made by adding rotary tables to CMMs. They both utilize the touch signal emitted by a probe when the tip ball comes into contact with the surface of a workpiece and records the probe coordinates at that time. The recorded coordinates are not valid points on the surface of the workpiece due to the signal delay, the inaccurate measurement coordinate system, and the radius of the probe, so the measured data need to be compensated further by the numerical algorithm.

The real face gears are the workpieces after processing, and they usually contain machining errors, which will affect the meshing performance. During the meshing process, the tooth surfaces of face gears and pinions will contact at a point at one moment, and the contact path will generate by connecting all contact points at every moment. Tooth surface deviations will alter the original tooth surface morphology, which will change the original contact path and further has a significant impact on the meshing performance of gear drives. In the industry, the meshing performance of real face gears can usually be analyzed by roll testing experiment, but this method is costly and timely. Therefore, some scholars have conducted tooth contact analysis for the real tooth surfaces, reconstructed with the measured data using CMMs or GMCs. Wang et al. [15] used the compensated measured data to reconstruct the tooth surface of face gears and conducted the digital tooth contact analysis using the proposed robust algorithm. Lin et al. [16] presented a least rotation angle method and the improved quad-tree search algorithm to compute the contact pattern and transmission error of a single flank fitted by the data measured on GMCs. Din et al. [17] proposed a collaborative manufacturing system for hypoid gears based on data-driven programming that the data are collected using CMMs, and the machine setting is modified by the tooth geometric and meshing performance.

The above researchers all adopted the contact metrology method. Although the contact metrology method, with the aid of compensation technology, can reach high accuracy, it has two disadvantages stated as follows.

(1) The border area of the tooth surface cannot be measured accurately since other locations would be touched. Moreover, the accuracy of the extrapolation method is insufficient when reconstructing the tooth surfaces. Hence, it is impossible to accurately determine whether edge contact occurs during the meshing of the real face gear drives. Edge contact refers to the meshing of the border areas of the tooth surface between face gears and pinions, which leads to stress concentration and meshing impact [18,19]. It will cause obvious vibration and noise during the meshing and reduces the service life of gears.
(2) The efficiency is low when measuring multiple teeth since every point needs to be contacted.

3D optical scanning, as a non-contact metrology method, has become a new trend in measuring the macroscopic morphology of surfaces with the emergence and development of computer vision [20,21,22]. Recently, this technique has been studied to inspect and analyze gear quality. Chen et al. [23] proposed an optical inspection method for tooth surfaces using the projection moire technique and measured the deviation based on the reference gear tooth profiles measured by CMM. Gonzalez-Perez et al. [24] performed tooth contact analysis of cylindrical gears reconstructed from point clouds. Urbas et al. [25,26] studied improved methods for optical measurement accuracy of spur gears. Three-dimensional (3D) optical scanning can be a concern by scholars due to the advantages as follows.

(1) As a non-contact metrology method, it is more efficient than the contact metrology method when measuring multiple teeth.
(2) The collected data using this method obtain the complete geometry of a tooth, including the top land surface, the bottom land surface, and both sides of the holistic tooth surface.

However, 3D optical scanning for measurement has not been widely used in the gear industry due to the low measurement accuracy. Its accuracy typically varies from 10 μm up to 100 μm [27,28,29,30]. The measurement errors come from two aspects. One is the systematic errors, and the other is the alignment errors between the measured workpiece and the design model. The former can only be reduced by upgrading the hardware specifications, while the latter can be reduced by alignment using the numerical algorithm. Thus, it is possible to achieve an accuracy of several microns with some numerical algorithms for compensation. Moreover, the previous research objects are cylindrical gears, while geometric characteristics of face gears are different, and the macroscopic morphology of tooth surfaces is extremely complex. 

In order to solve the problem of contact and non-contact metrology method and meet the increasing need for measuring holistic geometric information, a 3D optical scanning method is adopted for measuring the tooth surface of orthogonal face gears in this work, and the measurement solution is presented to ensure the measurement accuracy. In Section 2, the method of point cloud collection is introduced, and point clouds are processed by filtering and down-sampling. In Section 3, the measurement solution for tooth surfaces of face gears with 3D optical scanning is proposed to obtain the tooth surface deviations based on the alignment, and three-stage algorithm (3SA) is presented to align three important features between point clouds and the design model of face gears. In Section 4, the 3D modeling method is further investigated. An example is given to show the validity of the measurement solution with 3D optical scanning, and loaded tooth contact analysis (LTCA) for the real tooth surfaces of face gears by finite element method (FEM) is conducted to show the influence of the deviations on meshing performance in Section 5. 

## 2. Point Cloud Collection

**Figure 1:**
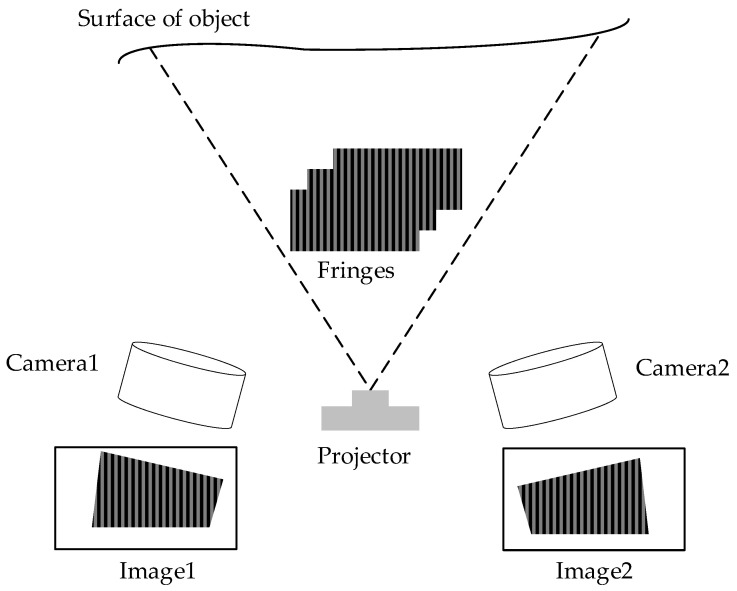
Fringe projection.

Point clouds containing the holistic geometry information of face gears can be collected at a rate of millions of points per second by 3D optical scanning. A way to implement 3D optical scanning is by binocular fringe projection method [31,32,33,34], as shown in Figure 1. The main principle is to project the fringes generated by the system onto the surface of objects in the corresponding order. Binocular cameras are applied to take images, and the original fringes will be found to be deformed due to the height change of the surface of objects. Then, the phase information of the surface of objects can be solved by the phase-shifting algorithm with the collected data of the deformed fringes. According to the phase information, the coordinate value of each point in the space can be calculated by triangulation, and then the point cloud data of the surface can be obtained. The binocular fringe projection method has been widely studied, and the detailed implementation methods will not be further described in the paper.

**Figure 2:**
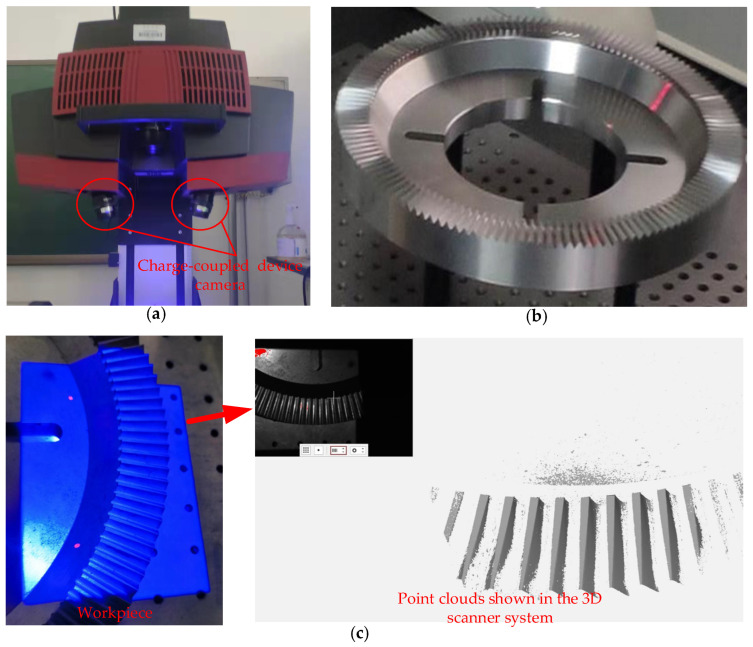
(**a**) ATOS 3D Scanner, (**b**) a face gear, and (**c**) the point cloud collection.

Currently, the available 3D scanning technology has been integrated into commercial equipment by some companies. Choosing the proper scanner equipment is a requirement for obtaining high-quality point clouds. Generally, the higher the spatial resolution and measurement accuracy of the equipment, the higher the quality of the collected point clouds of face gears. This work takes ATOS 3D Scanner to collect point clouds, as shown in Figure 2a. ATOS 3D Scanner is an industrial optical system with a charge-coupled device camera at both ends. The measured data by it can be transmitted to the computer, and the coordinate information of point clouds can be shown in the special software. The main system configuration is shown in Table 1.

When performing the operation, first ensure that the experiments are in a stable working environment, such as a stable light and warm-up environment, in which the measurement results of the 3D scanner are not affected by external factors. Then, the scanner is set up on the stationary support, and the settings of equipment are adjusted, such as lens position and aperture size. Subsequently, the scanner needs to be calibrated with the calibration plates. Through calibration, the system can accurately calculate the position of the scanning equipment relative to the scanning objects. Therefore, accurate calibration is a prerequisite for measurement accuracy. Moreover, the white developers need to be sprayed onto the face gear tooth surfaces to make them diffuse reflection for a better quality of point clouds. Since the bottom surface of face gears is the processing reference with high accuracy, face gears can be placed flat on the workbench when collecting point clouds, as shown in Figure 2b. Finally, the scanner system can be operated to take pictures of the workpieces, and the system would automatically obtain the coordinate data of the workpiece so that it obtains the point cloud data. In Figure 2c, a workpiece of face gear is being scanned by the 3D scanner, and the point clouds can be seen in the scanner system.

**Figure 3:**
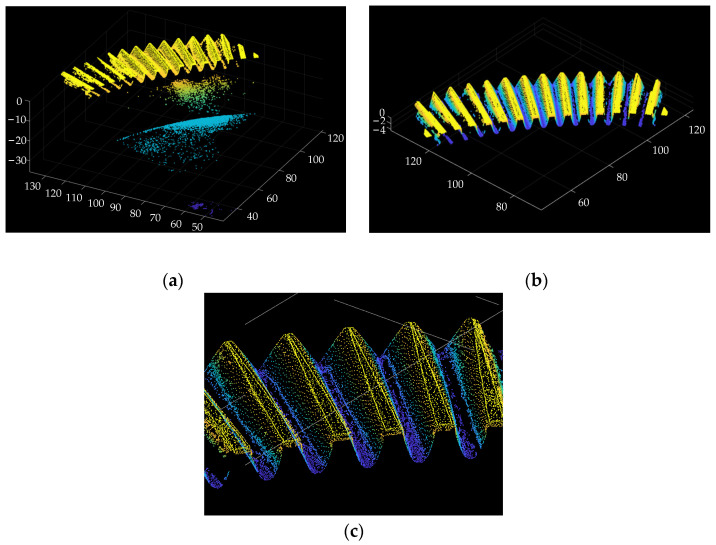
(**a**) Unprocessed point cloud, (**b**) filtering, and (**c**) down-sampling.

Subsequently, point clouds would require data processing. As shown in Figure 3a, the unprocessed point clouds will contain unnecessary point data. Only the points on the tooth surface are needed, and the other points should be removed. Here take the conditional filters to remove the outliers. Since the table is horizontal, the high area is the point cloud of tooth surfaces, and the low area is the point cloud of the conical surface and upper surface. When setting the appropriate *z*-value, the two different point clouds could be distinguished. The needed point cloud of tooth surfaces is shown in Figure 3b. If multiple point clouds need to be stitched together, the noise points will be generated, and further noise point reduction is needed with filter algorithms, such as the Gaussian filter [35]. Subsequently, the data volume of the point cloud can be reduced by the down-sampling method [36], as shown in Figure 3c. The different grid boxes in the space are divided, and a specific density of points in the grid is taken.

## 3. The Measurement Solution

**Figure 4:**
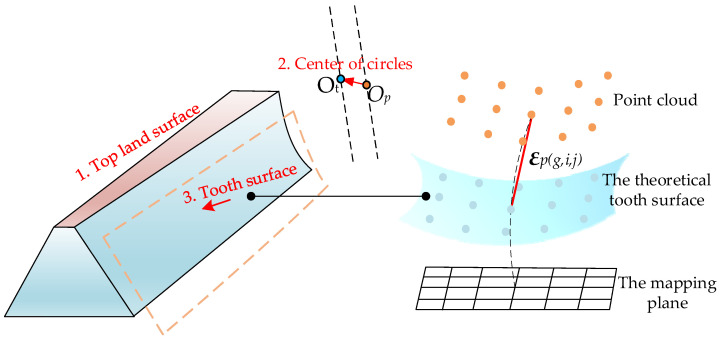
The three-stage alignment for top land surface, center of circles, and tooth surface.

The point cloud collected in Section 2 is in the measurement coordinate system (MCS), which is different from the design coordinate system (DCS). DCS is a fixed coordinate system determined in the design process of face gears, while MCS is the coordinate system set randomly in the measurement process. In order to obtain the tooth surface deviations of face gears, the point cloud containing the geometry information must be compared to the design model in the same coordinate system. The tooth surface deviation of one point can be represented as

(1) $$dev_{i}=\left(pm_{i}-pt_{i}\right)\cdot n_{i}$$

where ${pt}_{i}$ and $n_{i}$ are the coordinates and the unit normal of one point on the theoretical tooth surface in DCS, respectively. ${pm}_{i}$ is the measured point which is in the direction of $n_{i}$. Before computing the tooth surface deviations, alignment is required, and the geometric features between the point cloud and the design model are aligned, which is mathematically equivalent to transforming the coordinate system. The alignment is taken into three stages: the alignment for the top land surface, the center of circles, and the tooth surface, as shown in Figure 4, and 3SA is presented as follows.

(1) The first stage

The top land surface of the designed orthogonal face gears is a plane, which is parallel to the xoy plane in DCS, and the points on it are the same z-value. The collected points on the top land surface will not be the same z-value in MCS for the measurement and machining errors. Hence, the plane of the measured top land surface is fitted by the following equation as

(2) $$z_{\mathrm{ls}}=ax_{\mathrm{ls}}+by_{\mathrm{ls}}+c$$

The subscript ls represents the top land surface of the workpiece, and *a*, *b*, *c* are the coefficients. The solution can be obtained by solving the overdetermined systems of equations as

(3) $$A\cdot X=b\Leftrightarrow X={\left(AA^{T}\right)}^{-1}A^{T}b$$

where

(4) $$X=\left(\begin{array}{c} a\\ b\\ c \end{array}\right),A=\left[\begin{array}{ccc} x_{1} & y_{1} & 1\\ x_{2} & y_{2} & 1\\ x_{3} & y_{3} & 1\\ \vdots & \vdots & \vdots \\ x_{n} & y_{n} & 1 \end{array}\right],b=\left(\begin{array}{c} z_{1}\\ z_{2}\\ z_{3}\\ \vdots \\ z_{n} \end{array}\right)$$

$\left(x_{1}{,y}_{1}{,z}_{1}\right),\;\left(x_{2}{,y}_{2}{,z}_{2}\right),\;\left(x_{3}{,y}_{3}{,z}_{3}\right),\;\cdots ,\;\left(x_{n}{,y}_{n}{,z}_{n}\right)$ are the coordinates of the points on the top land surface of the workpiece. Then, the point cloud is translated along the *z*-axis as

(5) $$\Delta z=z_{\mathrm{ls}}-z_{t}$$

where $z_{t}$ is the value of the theoretical top land surface along the *z*-axis in DCS.

(2) The second stage

The center of circles O_t_ of the designed face gears is zero point, while the center of circles *O_p_* of the point cloud is set roughly. Hence, they are aligned by the pattern search technique [37,38]. The maximum and the minimum radius in the point cloud on the xoy plane are respectively *R_r_*_max_ and *R_r_*_min_, and their coordinates are respectively (*x_r_*_max_, *y_r_*_max_, *z_r_*_max_) and (*x_r_*_min_, *y_r_*_min_, *z_r_*_min_). The maximum and the minimum radius of the designed face gears are R_tmax_ and R_tmin_, respectively. The zero point in MCS is set on the inner side of the point cloud, so the initial value for the translation vector of the point cloud is set by the following equation as

(6) $$Ini\;=\;\left(\frac{\left(R_{r\max}-R_{\mathrm{tmax}}\right)}{R_{r\max}}x_{r\max},\frac{\left(R_{r\max}-R_{\mathrm{tmax}}\right)}{R_{r\max}}y_{r\max},0\right)$$

The following optimization objective is established as

(7) $$\min f=\mathrm{norm}\left(R_{r\max}-R_{\mathrm{tmax}},R_{r\min}-R_{\mathrm{tmin}}\right)$$

Then the optimal solution is searched as follows.

Step 1. Set the initial translation vector $q_{0}=Ini$, the initial step $\delta_{0}=1$, the contraction factor α=0.6, and the tolerance error ${\varepsilon =10}^{-6}$.

Step 2. Let ${p=q}_{k}\;\mathrm{and}\;j=1.$

Step 3. If $f\left({p+\delta }_{k}e_{j}\right)\;<\;f\left(p\right)$, ${p=p+\delta }_{k}e_{j}$ and turn to Step 5. Otherwise, turn to Step 4.

Step 4. If $\;f\left({p\;-\;\delta }_{k}e_{j}\right)\;<\;f\left(p\right)$, ${p=p\;-\;\delta }_{k}e_{j}$ and turn to Step 5. Otherwise, turn to Step 5.

Step 5. If j < n, j=j+1 and turn to Step 3. Otherwise, $q_{k+1}=p$ and turn to Step 6.

Step 6. If $\;f\left({\;q}_{k+1}\right)\;<\;f\left({\;q}_{k}\right)$, let ${p=q}_{k+1}+\left(q_{k+1}\;{-\;q}_{k}\right)$, $\delta_{k+1}{=\alpha \delta }_{k}$, k=k+1, j=1 and turn to Step 3. Otherwise, turn to Step 7.

Step 7. If $f\left({\;q}_{k}\right)\;<\;\varepsilon$, stop computation, and $q_{k}$ is the optimal solution. Otherwise, turn to Step 8.

Step 8. If $q_{k+1}{=q}_{k}$, let $\delta_{k+1}{=\alpha \delta }_{k}$, k=k+1 and turn to Step 2. Otherwise, let $q_{k+1}{=q}_{k}$, $\delta_{k+1}{=\delta }_{k}$, k=k+1 and turn to Step 2.

The optimal solution is obtained through the above steps, as

(8) $$q^{*}=\left(q_{1}{}^{*},q_{2}{}^{*},0\right)$$

where the superscript * represents the optimal solution. Then, the point cloud is translated according to the optimal solution as follows.

(9) $$\{\begin{array}{c} \Delta x=q_{1}{}^{*}\\ \Delta y=q_{2}{}^{*} \end{array}$$

(3) The third stage

In the third stage, the points after being processed in the second stage will be aligned with the theoretical tooth surface. The 5×9 grid on each side of the measured tooth surface of a tooth is selected randomly. The grids are translated and rotated around the *z*-axis. The transformed grids are represented as

(10) $$\left[\begin{array}{c} p_{\mathrm{tr}}\\ 1 \end{array}\right]=M_{tr}\cdot \left[\begin{array}{c} p_{\mathrm{mg}}\\ 1 \end{array}\right]$$

where

(11) $$p_{tr}=\left[\begin{array}{c} x_{\mathrm{tr}}\\ y_{\mathrm{tr}}\\ z_{\mathrm{tr}} \end{array}\right],p_{\mathrm{mg}}=\left[\begin{array}{c} x_{\mathrm{mg}}\\ y_{\mathrm{mg}}\\ z_{\mathrm{mg}} \end{array}\right],M_{\mathrm{tr}}=\left[\begin{array}{cccc} \cos\alpha & -\sin\alpha & 0 & dx\\ \sin\alpha & \cos\alpha & 0 & dy\\ 0 & 0 & 1 & dz\\ 0 & 0 & 0 & 1 \end{array}\right]$$

$p_{\mathrm{mg}}$ represents the points on the grids before the transformation, ***M***_tr_ represents the transformation matrix, and $p_{\mathrm{tr}}$ represents the point on the transformed grids. *α* is the rotation angle around the z-axis, and *dx*, *dy,* and *dz* are the translation values along the *x*-, *y*-, and *z*-axis, respectively. They are all chosen as the optimization variables. The reason for the second translation here is that the data processed in the first two stages will contain systematic errors from the top land surface and center of circles, so the tooth surfaces need to be further aligned to reduce the systematic errors.

Then, the rotation radius r and height h of each point of grids can be obtained after rotating the grids around the *z*-axis, and the points are mapped to the mapping plane. Every point on the theoretical tooth surface with the same rotation radius and height is calculated according to the tooth surface equation, as follows.

(12) $$\{\begin{array}{c} r_{\left(g,i,j\right)}=\sqrt{x_{\mathrm{tr}}{}_{\left(g,i,j\right)}{\left(var\right)}^{2}+y_{\mathrm{tr}}{}_{\left(g,i,j\right)}{\left(var\right)}^{2}}\\ h_{\left(g,i,j\right)}=z_{\mathrm{tr}}{}_{\left(g,i,j\right)}\left(var\right) \end{array},g=1,2;i=1,2,\cdots ,5;j=1,2,\cdots ,9$$

(13) $$\{\begin{array}{c} \sqrt{px_{\left(g,i,j\right)}{\left(uv_{1},uv_{2}\right)}^{2}+py_{\left(g,i,j\right)}{\left(uv_{1},uv_{2}\right)}^{2}}=r_{\left(g,i,j\right)}\\ pz_{\left(g,i,j\right)}(uv_{1},uv_{2})=h_{\left(g,i,j\right)} \end{array},g=1,2;i=1,2,\cdots ,5;j=1,2,\cdots ,9$$

where the different values of the subscript *g* represent the grid on the different tooth surfaces of a tooth. The subscript *i* and *j* represent the point number on the grid. $\left({px}_{\left(g,i,j\right)}{,py}_{\left(g,i,j\right)}{,pz}_{\left(g,i,j\right)}\right)$ represents the coordinate of the point on the theoretical tooth surface. ***var*** represents the unknown variables (*α,dx,dy,dz*). (*uv*_1_,*uv*_2_) represents two unknown variables of the tooth surface equation. The tooth surface equation of face gears has been derived in our previous literature [39], and here it is not stated in detail. The vector between the point $p_{\mathrm{tr}}$ on the transformed grid and the corresponding theoretical point $p_{\mathrm{tp}}$ can be yielded as

(14) $$\varepsilon_{p\left(g,i,j\right)}=p_{\mathrm{tr}\left(g,i,j\right)}\left(var\right)-p_{\mathrm{tp}\left(g,i,j\right)}\left(var\right)$$

In order to achieve the best alignment between the transformed grid and the theoretical tooth surface, a nonlinear least square minimization optimization is established, and it is represented as

(15) $$minf_{\varepsilon }\;=\;\frac{1}{2}\sum_{g,i,j}\varepsilon_{p\left(g,i,j\right)}{\left(var\right)}^{T}\varepsilon_{p\left(g,i,j\right)}\left(var\right)$$

The above over-determined nonlinear equation is expanded by second-order Taylor to transform it into a trust region problem, as

(16) $$\min O_{\varepsilon k}\left(d\right)=f_{\varepsilon k}+\nabla f_{\varepsilon k}{}^{T}d+\frac{1}{2}d^{T}\nabla^{2}f_{\varepsilon k}d$$

where ***d*** is the optimal step of the iteration. $f_{\varepsilon k}$ is the value of $f_{\varepsilon }$ in the *k*th iteration. $\nabla f_{\varepsilon k}$ and $\nabla^{2}f_{\varepsilon k}$ are the first and second gradient, respectively. For convenience, $\nabla f_{\varepsilon k}$ is marked as ***g****_k_*. Since the calculation for $\nabla^{2}f_{\varepsilon k}$ is too complex, the approximate Hessian matrix $B_{k}$ is used to replace, as

(17) $$B_{k}=J_{k}{}^{T}J_{k}$$

where $J_{k}$ is the Jacobi matrix. Subsequently, the trust region dogleg method is used to solve the optimization function. The optimization problem is represented as

(18) $$\begin{array}{l} d=\arg\min f_{\varepsilon k}+g_{k}{}^{T}d+\frac{1}{2}d^{T}B_{k}d\\ s.t.\left\|d\right\|\;\leq \Delta_{k} \end{array}$$

where $\Delta_{k}$ is the trust region radius. The solution to Equation (18) can be given as

(19) $$d^{*}=\{\begin{array}{c} \tau \cdot C,0\leq \tau \leq 1\\ -C+\left(\tau -1\right)\cdot \left(GN+C\right),1<\tau \leq 2 \end{array}$$

where

(20) $$C=-\frac{g_{k}{}^{T}g_{k}}{g_{k}{}^{T}B_{k}g_{k}}g_{k}$$

(21) $$GN=-{\left(B_{k}\right)}^{-1}g_{k}$$

***C*** is the Cauchy step, and ***GN*** is the Gauss-Newton step. τ can be solved by the following scalar quadratic equation as

(22) $${\left\|-C+\left(\tau -1\right)\cdot \left(GN+C\right)\right\|}^{2}=\Delta_{k}$$

The ratio $\rho_{k}$ is used to measure the approximation between Equations (15) and (18), as follows.

(23) $$\rho_{k}=\frac{f_{\varepsilon }\left({var}_{k}\right)-f_{\varepsilon }\left({var}_{k}+d\right)}{O_{\varepsilon k}\left(0\right)-O_{\varepsilon k}\left(d\right)}$$

where ${var}_{k}$ is the value of optimization variables in the *k*th iteration. When $0.9{\;<\;\rho }_{k}\;\leq \;1,$ the approximation is well and $\Delta_{k}$ is increased. When $0{.1\;<\;\rho }_{k}\;\leq \;0.9,$ $\Delta_{k}$ remains the same. When $\rho_{k}\;\leq \;0.1,$ $\Delta_{k}$ is reduced.

When the optimal solution is solved, the point cloud can be transformed with

(24) $$M_{\mathrm{tr}}{}^{*}=\left[\begin{array}{cccc} \cos\alpha^{*} & -\sin\alpha^{*} & 0 & dx^{*}\\ \sin\alpha^{*} & \cos\alpha^{*} & 0 & dy^{*}\\ 0 & 0 & 1 & dz^{*}\\ 0 & 0 & 0 & 1 \end{array}\right]$$

## 4. 3D Modelling

To better visualize the tooth surface deviations using 3D software and further carry out contact simulation by FEM [40,41,42,43], the 3D model of real five-pairs-of-teeth face gears needs to be modeled. For complex surface reconstruction, non-uniform rational b-spline (NURBS) [44,45,46] is the common modeling method. However, the disorderly scattered points make it difficult to reconstruct the face gear tooth surface accurately. Therefore, it is necessary to determine the regularly arranged points in the range of the tooth surface and label the points with serial numbers. 

**Figure 5:**
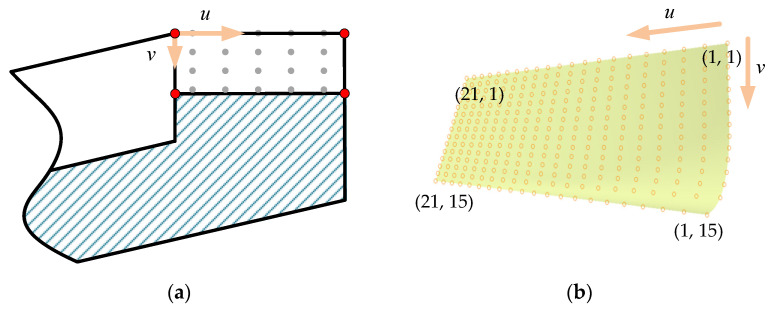
(**a**) The regularly discrete points on the theoretical tooth surface, and (**b**) the point cloud with numbered points.

As shown in Figure 5a, the theoretical points arranged equidistantly within the range of tooth surface are calculated first according to the equation of face gear tooth surface [39], and (a,b) is used to record the sequence number of points. The nearest points ***cp*** from the point cloud are found by calculating the minim Euler distance E from the theoretical points **tp**_(a,b),_ and ***cp*** will be numbered as same as **tp**_(a,b)_, as follows.

(25) $$\min E={\left\|cp_{\left(a,b\right)}-tp_{\left(a,b\right)}\right\|}_{2}$$

The point cloud with numbered points is obtained, as shown in Figure 5b. Subsequently, the tooth surface is represented as a bicubic NURBS surface, as

(26) $$S\left(u,v\right)=\frac{\sum_{i=0}^{m}\sum_{j=0}^{n}N_{i,3}\left(u\right)N_{j,3}\left(v\right)\omega_{i,j}d_{i,j}}{\sum_{i=0}^{m}\sum_{j=0}^{n}N_{i,3}\left(u\right)N_{j,3}\left(v\right)\omega_{i,j}}$$

where

(27) $$\left\{ \begin{array}{l} N_{i,0}\left(u\right)=\{\begin{array}{c} 1,u_{i}\leq u<u_{i+1}\\ 0,\mathrm{otherwise} \end{array}\\ N_{i,p}\left(u\right)=\frac{u-u_{i}}{u_{i+p}-u_{i}}N_{i,p-1}\left(u\right)+\frac{u_{i+p+1}-u}{u_{i+p+1}-u_{i+1}}N_{i+1,p-1}\left(u\right),p=1,2,3 \end{array}\right.$$

(28) $$\left\{ \begin{array}{l} N_{j,0}\left(v\right)=\{\begin{array}{c} 1,v_{j}\leq v<v_{j+1}\\ 0,\mathrm{otherwise} \end{array}\\ N_{j,p}\left(v\right)=\frac{v-v_{j}}{v_{j+p}-v_{j}}N_{j,p-1}\left(v\right)+\frac{v_{j+p+1}-v}{v_{j+p+1}-v_{j+1}}N_{j+1,p-1}\left(v\right),p=1,2,3 \end{array}\right.$$

*u* is along the radius of the face gear, and *v* is along the depth of the tooth. *m* and *n* are the number of control vertex of *u* and *v*, respectively. Set *m* = 21 and *n* = 15. *ω_i,j_* is the weight factor. *N_i_*_,3_ and *N_j_*_,3_ are the basis functions of *u* and *v*, respectively. *d_i,j_* is the control vertex.

**Figure 6:**
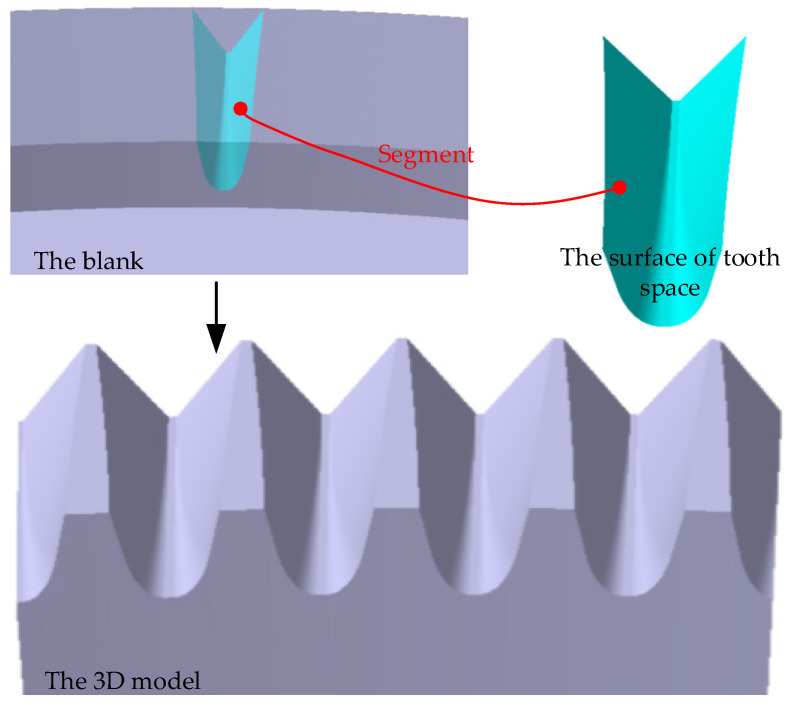
A 3D model of real five-pairs-of-teeth face gear.

According to Equation (26), a tooth surface can be reconstructed, and a tooth space surface is composed of two tooth surfaces. Another tooth surface can be reconstructed by the same method. Then, the tooth space model can be obtained by Boolean operation between the tooth space and the face gear blank. Further, the 3D model of real five-pairs-of-teeth face gears can be obtained by segmenting the blank with the other tooth space surfaces, as shown in Figure 6.

## 5. Example and Discussion

**Figure 7:**
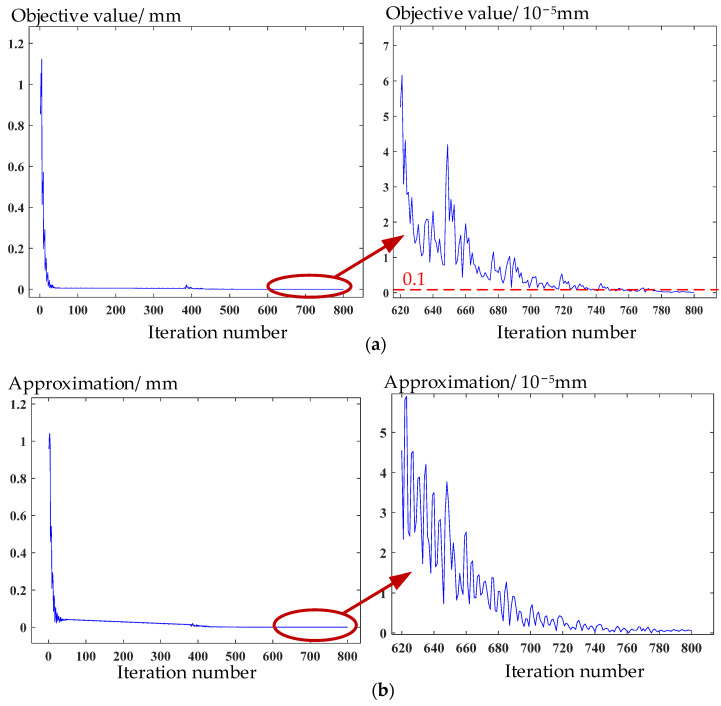
Convergence curves of (**a**) objective value and (**b**) iterative solution in the second stage of the algorithm.

**Figure 8:**
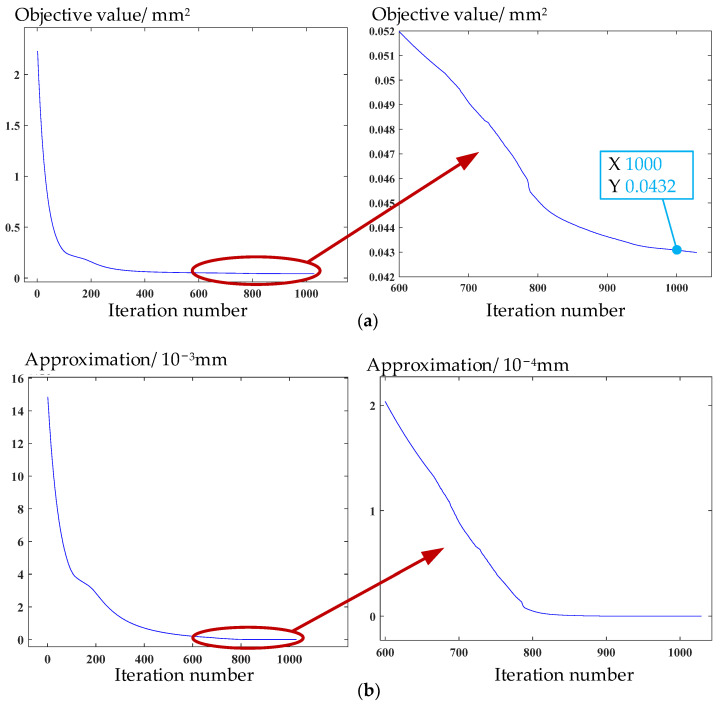
Convergence curves of (**a**) objective value and (**b**) iterative solution in the third stage of the algorithm.

Experiment with point cloud collection on the workpiece of face gear machined by grinding is conducted as stated in Section 2, and the parameters of a face gear are shown in Table 2. After being downsampled, the point cloud with 40,377 points is obtained. Subsequently, the point cloud is aligned using the measurement solution proposed in Section 3. In Figure 7a, the curve of objective value versus iteration number shows that the objective value of Equation (7) can reach the set accuracy at the 775th iteration. Then, the approximation of the solution at each iteration to the solution at the 775th iteration is defined by the 2-norm distance between them in the parametric space. The curve in Figure 7b shows that the solution tends to converge. Figure 8a shows that the change of the objective value of Equation (15) is extremely little after the 1000th iteration, reaching below 10^−4^ mm^2^. Figure 8b shows that the approximation between the solution at each iteration and the solution at the 1000th iteration is convergent. The convergence accuracy achieved at the 1000th iteration has almost no effect on the measurement results requiring micron-level accuracy. The above shows that 3SA has convergence characteristics.

As shown in Table 3, the objective value after the second stage of 3SA reaches 9.537 × 10^−7^ mm, which is a great improvement compared with 0.121 mm at the beginning, and the objective value of the tooth surface in the third stage increases from 2.23 mm^2^ to 0.0457 mm^2^.

**Figure 9:**
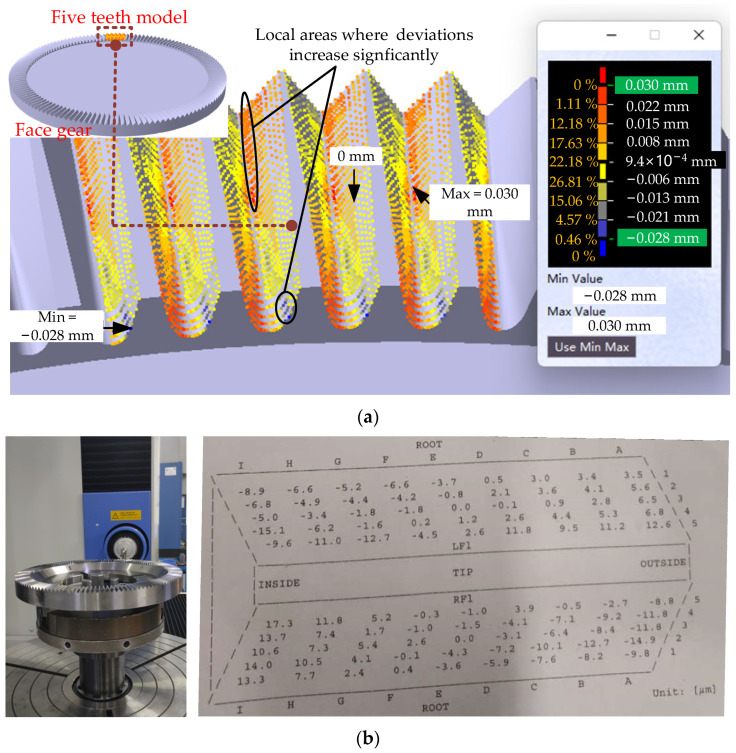
(**a**) Holistic deviation of the tooth surfaces obtained by the proposed method and (**b**) the measurement report of GMC.

The tooth surface reconstructed from the point cloud is compared with the theoretical tooth surface in the CATIA, as shown in Figure 9a. When viewing from top to bottom, the left side of the tooth is defined as the left tooth surface, and the right side is the right tooth surface. The result is stated as follows.

(1) Maximum deviation, not more than 0.030 mm, is on the border areas. The deviations above 0.022 mm are extremely few, and most are below 0.022 mm.

(2) The tooth surface deviations are inclined. The deviations of the inner side on the left tooth surface are negative, the deviations of the middle area trend to 0, and the deviations of the outer side are positive. It indicates that the inner side is overcut, and the outer side is undercut. While the deviations of the inner side on the right tooth surface are positive, and the deviations of the outer side are negative, which indicates that the inner side is undercut, and the outer side is overcut.

(3) The deviation trends of five teeth are almost the same.

The probable reason for the above result of deviation is that there is an angle error in the installation of the tool. Compared the measurement result of the proposed method with that of GMC shown in Figure 9b, the trend of deviation distribution is consistent. Although the maximum deviation is larger, it is distributed in the border areas that cannot be measured by GMC. The error between tooth surface deviations obtained by GMC and the proposed method is less than 7 μm, which meets the measurement requirements for most face gears. It demonstrates that the measurement solution with 3D optical scanning is valid.

Moreover, the measurement solution proposed in the paper can show deviations in the border areas and more details of deviations. For example, the deviations increase significantly in some local areas shown in Figure 9a, which may be caused by the profile error of the worm wheel.

**Figure 10:**
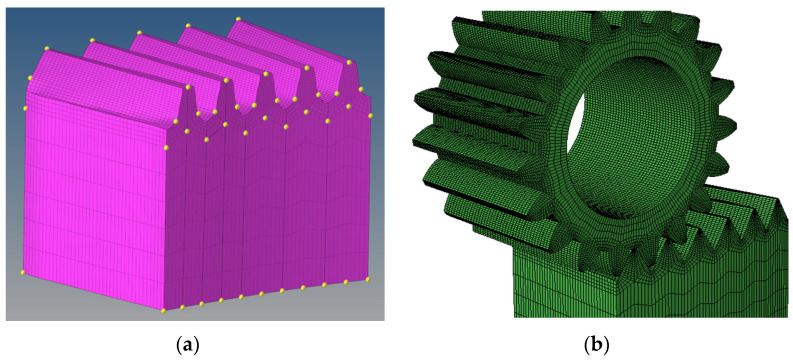
FEM: (**a**) Mesh generation, (**b**) the finite element model of real five-pairs-of-teeth face gear drive.

In order to further study the effect of the deviations on the contact performance of the face gear drives in this example, the 3D model of real five-pairs-of-teeth face gear is obtained according to Section 4, and LTCA is conducted further by FEM. It should be noted that the model here contains the tooth surface deviations, and the deviations of different teeth are a little different, so the previous method cannot be used to generate the mesh. In the previous mesh generation method, the finite element meshes of a tooth model are generated first, and the finite element meshes of other teeth models are obtained by rotation. While the five teeth model contains tooth surface deviations, the finite element meshes of every tooth needs to be generated, and all finite element meshes are combined, as shown in Figure 10a. Due to the proven processing technology of the pinions, the designed model of the pinion without deviation in this example is applied, and the face gear drive is assembled, as shown in Figure 10b. A torque load of 160,000 N·mm is applied to the pinion to conduct the simulated meshing process of the real face gear drive.

**Figure 11:**
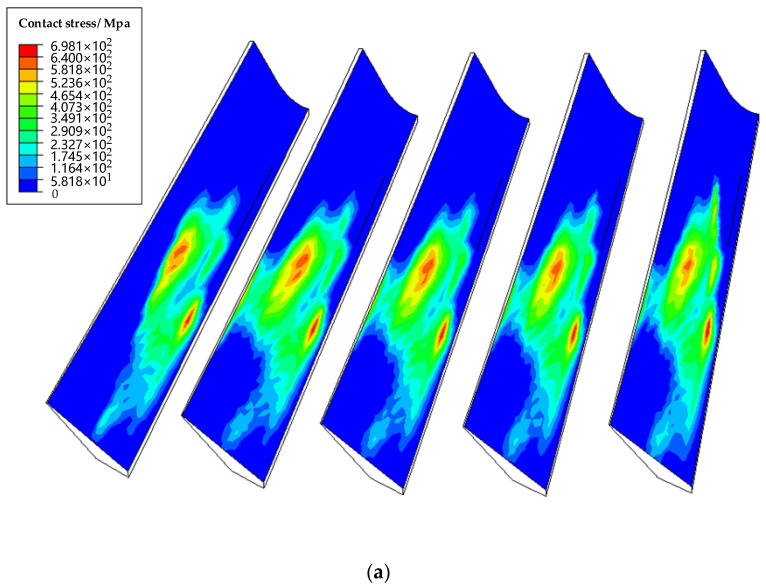

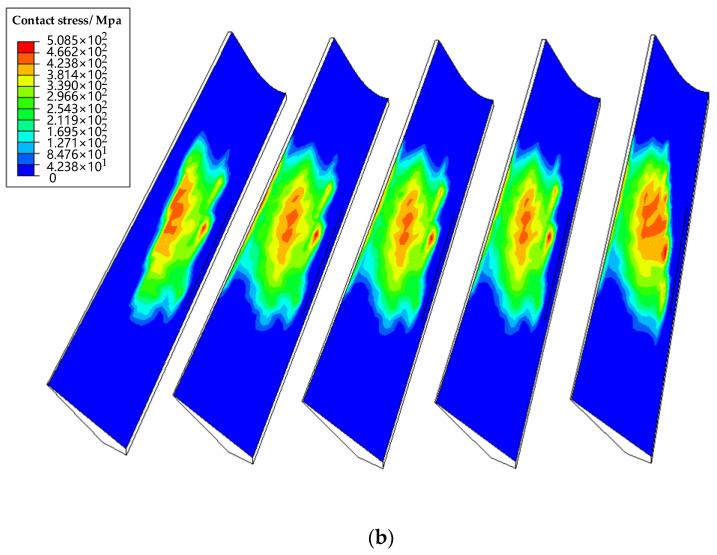
The simulation results for the contact pattern and contact stress of (**a**) the real face gear drive and (**b**) the designed face gear drive.

**Figure 12:**
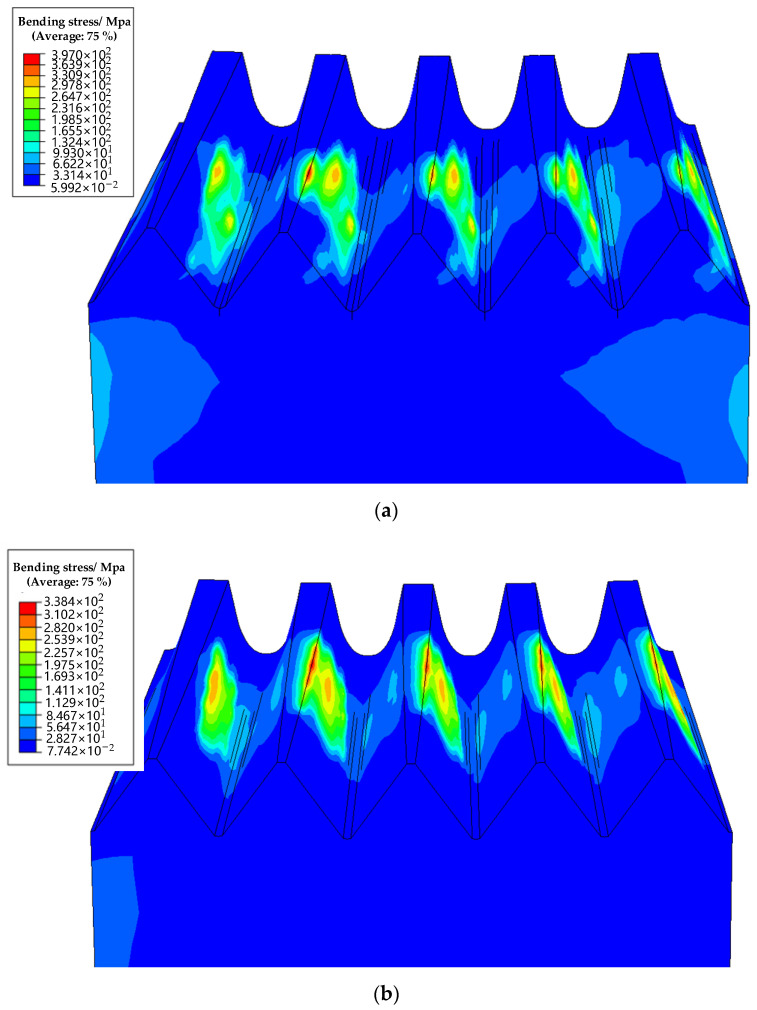
The simulation results for the bending stress of (**a**) the real face gear drive and (**b**) the designed face gear drive.

The result of combining all the simulation frames is shown in Figure 11a,b and Figure 12a,b. The first and fifth teeth are the incoming and outgoing teeth of the model, respectively, so they are excluded for comparison. Figure 11 shows the contact pattern of real face gear tends to the area with the maximum tooth surface deviation and the outer side, which will cause edge contact. The maximum contact stress of real face gear is about 200 Mpa, larger than that of designed face gear. Moreover, the maximum contact stress of real face gear occurs in the border areas, which is the area with the maximum deviation meanwhile. The reason is that the “bulge” formed by the positive deviations of the tooth surface in this example will make contact earlier than the original contact points during the meshing of face gear drives. Further, it destroys the original contact path, and the “bulge” bears more contact load so that the contact stress is more concentrated in the border areas.

From Figure 12, the root bending stress of real face gear is 330.9 Mpa, while that of designed face gear is about 200 Mpa. The root bending stress increased by about 0.65 times, which will greatly affect the service life of face gear. The deviation difference between the different teeth has almost no influence on the meshing of face gear drive in this example.

The result of LTCA shows that the deviations in the border areas cause the change of contact pattern, contact stress, and root bending stress. The specific influence in this example is stated as follows.

(1) Edge contact occurs due to the deviations. The real face gear drive cannot make smooth contact as the designed model, and the tooth surfaces of the face gear and pinion collide with each other, which will cause vibration and noise. Hence, the stability of the transmission of the face gear drive will be reduced.

(2) Edge contact causes the contact stress concentrated in the border areas, and contact stress is increased by approximately 0.4, resulting in more easily occurring tooth surface pitting failure. The root bending stress is increased by approximately 0.65, resulting in more easily occurring tooth root breakage failure. The increase in contact stress and root bending stress will reduce the service life of the face gear.

Therefore, further processing should be conducted for the areas with large positive deviations, especially the border areas of the tooth surface. A simple method is to use an end mill cutter to mill the border areas of the tooth surface. The more flexible and practical method is to correct the settings of the grinding machine tool to reduce the machining errors or to modify the profile of the grinding tool to process the higher-performance tooth surface according to the measurement result of the proposed method.

## 6. Conclusions

In this paper, the measurement solution with 3D optical scanning is proposed to measure the tooth surfaces of orthogonal face gears. Aiming at the problem that the contact metrology method cannot measure the border areas of tooth surfaces and inefficiently acquires the huge number of points, this paper adopts the 3D optical scanning method to acquire point clouds, and the measurement solution is proposed to ensure measurement accuracy for the tooth surfaces. A 3SA is presented to align the features between point clouds and the designed face gears, including top land surfaces, centers of circles, and tooth surfaces. Moreover, the 3D modeling method is studied by reconstructing the real tooth surface with the points after being numbered. An example is given to show that the measurement solution with 3D optical scanning is valid. With the comparison, the proposed measurement solution can show the deviations in the border areas and more details that the previous measurement method could not reveal. LTCA is further conducted to study the meshing performance of the real tooth surface. The results of LTAC show that the deviations in the border areas cause edge contact, which will significantly reduce the contact strength and bending strength of real face gear. Summarily, the proposed measurement solution can provide a more reliable base for correction processing.

The proposed measurement solution can meet the measurement required for the tooth surfaces of face gears applied in automobile and other industries. In the future, the numerical algorithm of precise multi-point cloud splicing will be studied to measure face gears with all teeth, and the measurements of more precision face gears that can be applied to the aerospace industry will be conducted.

## Figures and Tables

**Table 1:** The configuration of ATOS 3D Scanner.

System	ATOS 3D Scanner
Camera pixels	2 × 3296 × 2472 pixels
Measuring volumes	${38\;\times \;29\;\times \;15\;–\;2000\;\times \;1500\;\times \;1500\;\mathrm{mm}}^{3}$
Point spacing	0.01–0.61 mm
Working distance	490–2000 mm
Projected light source	Structured blue light
Operating Temperature	5–40 °C

**Table 2:** The parameters of a face gear.

Parameters	Values
Shaper teeth number N*_s_*	22
Pinion teeth number N_1_	19
Face gear teeth number N_2_	142
Module m	1.95 mm
Pressure angle of the rack cutter α	25°
Shaft angle γ*_m_*	90°
Inner radius L_1_	128 mm
Outer radius L_2_	152 mm

**Table 3:** The comparison of objective value before and after computing in 3SA.

		Second Stage	Third Stage
Objective value	Before	0.868 mm	2.23 mm^2^
	After	9.537 × 10^−7^ mm	0.0043 mm^2^

## Data Availability

Not applicable.
